# Clinical characteristics of children infected with SARS-CoV-2 in Italy

**DOI:** 10.1186/s13052-021-01045-0

**Published:** 2021-04-15

**Authors:** Isabella Tarissi De Jacobis, Rosa Vona, Camilla Cittadini, Alessandra Marchesi, Laura Cursi, Lucrezia Gambardella, Alberto Villani, Elisabetta Straface

**Affiliations:** 1 Internal Care Department, General Pediatric and Infectious Disease Unit, Bambino Gesù Children’s Hospital, Rome, Italy; 2 Biomarkers Unit, Center for Gender-Specific Medicine, Istituto Superiore di Sanità, Viale Regina Elena 299, 00161 Rome, Italy

**Keywords:** Sex differences, COVID-19, Inflammation, Thrombocytosis, Pediatric population

## Abstract

**Background:**

Since December 2019 coronavirus disease (COVID-19) emerged in Wuhan and spread rapidly worldwide. Despite the high number of people affected, data on clinical features and prognostic factors in children and adolescents are limited. We propose a retrospective study aimed to evaluate clinical characteristics of children infected with SARS-CoV-2 in Italy.

**Methods:**

A pediatric population admitted with COVID-19 to Bambino Gesù Children’s Hospital of Rome (Italy) in the period from the end of February to July 2020 has been studied. Medical history, comorbidities, symptoms and laboratory findings were obtained from patients’ electronic medical records.

**Results:**

In 66 patients (35 males and 31 females) we found that: i) fever and cough were the dominant symptoms, while vomit and convulsions were rare symptoms; and ii) all ages of childhood were susceptible to COVID-19. Furthermore, we found that, compared to females, males with COVID-19, although not significantly, had higher values of inflammatory markers such as C-reactive protein (CRP) and ESR. Conversely, we found that COVID-19 positive females were older than males and required more days of hospitalization. Both males and females COVID-19 positives had procalcitonin values within the normal range and D-Dimer values slightly higher than the normal range. With regard to this latter marker, the value measured in females, although not significant, was higher than that measured in males. Interestingly, the presence of leukopenia was found in both sexes.

**Conclusions:**

Compared to the adults we found that COVID-19 infection in children is a non-severe inflammatory disease in both males and females. In any case, many detailed studies should be conducted.

## Background

Since mid-December 2019, an infection caused by a new type of coronavirus (SARS-COV-2) emerged in Wuhan (Hubei Province, China) and spread rapidly worldwide. The emerging SARS-COV-2 is a beta coronavirus that can cause COVID-19, officially named by the World Health Organization (WHO) on February 11, 2020. This virus is highly contagious and can be transmitted by an infected person or an asymptomatic carrier through respiratory droplets, tear fluid and close contacts. The incubation period is variable. It has been estimated that the median incubation period is 5.1 days and that 97.5% of infected patients will develop symptoms within 11.5 days of infection.

Despite the high number of people affected, data on clinical features and prognostic factors in children and adolescents are limXited. Children are part of a very special group. Similarly to the SARS-COV 2002–2003 epidemic [1, 2], pediatric COVID-19 appears to be mild or asymptomatic [3, 4]. Children become less ill than adults and most of them contract the infection mainly through close contact with their parents or other family members with COVID-19. Many children infected with SARS-COV-2 manifest a mild disease that often does not require hospitalization. Compared to adults, children have a lower chance of developing interstitial pneumonia, one of the most serious complications of the infection, which in the advanced form requires hospitalization in intensive care. As for the adults, the presence of congenital heart disease, lung and airway disease, malnutrition, and cancer makes children more susceptible to COVID-19.

There are several hypotheses on the mechanisms underlying the lower susceptibility of children to COVID-19 infection than adults: i) a more efficient immune response due to the stimulation given by typical age vaccinations; ii) a lower expression of the angiotensin-converting enzyme 2 (ACE2) receptor to which the virus would bind to enter cells [5]; iii) an “immaturity” of the ACE2 receptors, which makes it difficult for the virus to enter the body [6]; and iv) external factors (before the lockdown, children were less likely than adults to visit places that could have facilitated the spread of the virus, such as railway stations and airports) [7].

In children with COVID-19, fever and cough are the most common clinical manifestations, sometimes accompanied by fatigue, myalgia, nasal congestion, sneezing, sore throat, headache, dizziness, vomit and abdominal pain. Moreover, some children do not manifest fever, but only cough or diarrhoea, or they may be asymptomatic. The latter, even if they do not manifest symptoms, may play significant roles in the transmission of COVID-19 in the community.

Italy was one of the European countries most affected by the COVID-19 pandemic. By 16 April 2020, 1123 children, up to 9 years of age, and 1804 adolescents, aged between 10 and 19 years old were tested positives for COVID-19 [8].

In Italy most of the data on COVID-19 pediatric patients derive from a multicentre study promoted by the Italian Society of Paediatric Infectious Diseases (SITIP), within the Italian Society of Paediatrics (SIP). In this study 168 children aged 1 day to 17 years, 94 (55.9%) males and 74 (40.1%) females, with confirmed COVID-19 were analysed [9]. 65.1% of these children were hospitalized: of these, only 17 (15.5%) were sent to the hospital after seeing a paediatrician or family doctor. Moreover, 5.9% of children documented co-infections with other viruses such as respiratory syncytial virus, rhinovirus, Epstein-Barr virus, influenza A virus and a non-SARS coronavirus. Bacterial co-infection with *Streptococcus pneumoniae* has also been documented. Pre-existing chronic pathologies, such as chronic lung diseases (*n* = 7), congenital malformations or complex genetic syndromes (*n* = 14), cancer (*n* = 4), epilepsy were found in 33 children. Moreover, gastrointestinal (*n* = 2) or metabolic (*n* = 1) disorders were found. Among these patients 4 were immunosuppressed and 3 immunocompromised. The hospitalization rate was similar between children with and those without co-morbidity.

Studies have reported a higher incidence of COVID-19 in males than in females in the adult population [10]. This study is aimed to evaluate clinical characteristics of children infected with SARS-CoV-2 in Italy.

For this purpose, 66 patients admitted to Bambino Gesù Children’s Hospital of Rome (Italy) in the period from the end of February to July 2020 were analysed.

## Methods

### Study design and participants

Sixty-six patients (35 males and 31 females), admitted with COVID-19 to Bambino Gesù Children’s Hospital of Rome (Italy) in the period from the end of February to July 2020, were enrolled in this retrospective cohort study. Mean age of patients was 6.8 years (range ≤ 1–18 years).

All patients had contracted the infection from their parents and they were hospitalized because showed signs and symptoms such as fever, cough, vomit, diarrhea, convulsions, headache or pneumonia. Fourteen patients were asymptomatic (7 males and 7 female), but they were hospitalized and monitored until they tested negative at the swab (Table 1).

**Table 1:** Features of patients at admission

Characteristics	Males (***n*** = 35)	Females (***n*** = 31)
**Age, median (range)-years**	5.98 (range ≤ 0.1–14.8)	8.1 (range ≤ 0.2–16)
**Hospitalization, median (range)-days**	7.24 (range 3–21)	9.50 (range 3–22)
**Signs and symptoms**		
**Asyntomatic**	7 (20%)	7 (22.5%)
**Fever**	13 (37%)	12 (38%)
**Cough**	6 (17%)	7 (22.5%)
**Vomit**	1 (2.8%)	2 (6.4%)
**Diarrhea**	5 (14%)	3 (9.6%)
**Convulsions**	1 (2.8%)	4 (12.9%)
**Headache**	4 (11%)	3 (9.6%)
**Coinfections**	3 Rhinovirus (8.6%) 1 Campylobacter (2.8%) 1 Enterovirus (2.8%) 1 *E. coli* (2.8%)	2 EBV (6.4%) 1 HHV6 (3.2%)
**Pneumonie**	2 (5.7%)	0

For age and hospitalization days both median and range of values (from the lowest value to the highest value) detected in male and female patients are shown

For signs and symptoms both patients number and percentage of patients are shown

The study was performed in accordance with Good Clinical Practice and the Declaration of Helsinki principles for ethical research. Ethics approval and written informed consent were waived due to the rapid emergence of this infectious disease. Three researchers and a physician collected and reviewed the data. Medical history, underlying comorbidities, symptoms and laboratory findings both at admission and during hospitalization, were obtained from patients’ electronic medical records. The date of disease onset was defined as the day when the symptoms were noticed.

### Laboratory measurements

All patients underwent nasopharyngeal, eye, urine and stool swab. The presence of SARS-CoV-2 in respiratory specimens was detected by real-time reverse transcription (RT-PCR) methods. Analyses by gene amplification reaction and Real Time RT-PCR were also carried out to exclude evidence of other viral infections, including influenza, respiratory syncytial virus, avian influenza, para-influenza, adenovirus and rhinovirus. Routine bacterial and fungal examinations were also performed. Moreover, EBV and herpes virus (HHV6) infections were routinely screened and detected by using a test that identifies antibodies to EBV and HHV6 in the blood.

Data include all paediatric patients in whom COVID-19 was documented by at least one nasal/ pharyngeal swab specimen positive for SARS-CoV-2 nucleic acid using RT-PCR assay.

### Statistical analysis

To compare medians and range of values between two groups we used the Mann-Whitney’s U test. The level of significance was determined at *p* <  0.05.

## Results

### Features of patients at admission

At admission patients presented: fever (25 patients), cough (13 patients), vomit (3 patients), diarrhoea (8 patients), convulsions (5 patients) and headache (7 patients). Moreover, 14 patients were asymptomatic and 9 patients had co-infections: 3 with Rhinovirus, 1 with Campylobacter, 1 with Enterovirus, 1 with *Escherichia coli*, 2 with Epstein-Barr virus (EBV) and 1 with Herpes virus (HHV6) (Table 1). Some analyzed patients had a history of pneumonia, bronchiolitis, asthmatic bronchitis, gastroenteritis and convulsions. Furthermore, at admission only 23 patients (15 males and 8 females) presented bronchospasm and underwent chest x-ray. In 3 males and 5 females, a modest thickening of the peri-broncho-vascular interstitium was found.

Interestingly, analyzing medical records we found that in pediatric population with COVID-19: i) females, although not significantly, were older than males and required more days of hospitalization (Table 1); ii) males had CRP and ESR values higher than the normal range, but no significant gender differences were detected (Table 2); iii) for both sexes, fibrinogen and procalcitonin values were in the normal range and were not different between the two sexes; and iv) for both sexes the D-Dimer values were higher than the normal range. Although not significant, the value measured in females was higher than those measured in males. Moreover, we found that, lactate dehydrogenase (LDH) values were higher than the normal range in both males and females and were significantly higher in females than male (*p* = 0.05). Conversely, platelet number was in the normal range, in both sexes (Table 2). For parameter such as WBCs and RBCs we evaluated the data take into account of the normal ranges according to age of patients. Regarding WBCs, for all ages considered, the number of cells was below the normal range (these data highlight the presence of leukopenia in both males and females). Conversely, for every age group, values higher than the normal range were measured in both males and females. Significant gender differences were detected in patients from 0 to 1 year (*p* = 0.0132) (Table 2).

**Table 2:** Laboratory findings of patients with COVID-19

Markers	Normal range	Males (***n*** = 35)	Females (***n*** = 31)	***P*** values
**CRP (mg/dL)**	< 0.5	0.66 (range 0.03–5)	0.28 (range 0.03–3.39)	0.605
**ESR (mm/h)**	0–15	15.56 (range 4–26)	9.13 (range 4–21)	0.092
**Fibrinogen (mg/dL)**	150–400	370 (range 217–566)	340 (range 212–540)	0.4813
**Procalcitonin (ng/ml)**	< 0.5	0.08 (range 0.02–1.16)	0.29 (range 0.02–2.28)	0.6791
**D-Dimer (μg/ml)**	0–0.57	0.75 **(**range 0.27–3.35)	1.83 (range 0.27–19.69)	0.6228
**LDH (U/L)**	60–170 (range 1–10 years)	266,6 **(**range 188–365)	317.5 **(**range 194–453)	*0.0499
**PLT (10**^**3**^**/uL)**	150–450	282.40 (range 135–445)	247.96 (range 170–352)	0.1072
**WBCs (10**^**3**^**/uL)**	0–3 years (10–25)	3.9 (range 1.9–6.8)	5.5 (range 2.7–13)	0.2642
**WBCs (10**^**3**^**/uL)**	4–7 years (6–15)	3 (range 0.5–6.1)	3.7 (range 2.1–6)	0.9307
**WBCs (10**^**3**^**/uL)**	8–12 years (4.5–13)	2.2 (range 1.2–3.6)	2.9 (range 1.4–5.4)	0.6806
**WBCs (10**^**3**^**/uL)**	13–18 years (4–10)	1.2 (range 1–1.6)	1.5 (range 0.3–3.2)	0.6508
**RBCs (10**^**6**^**/uL)**	0–1 year (3.2–4.8)	6.5 (range 2.9–11.2)	10.9 (range 8.2–1.7)	*0.0132
**RBCs (10**^**6**^**/uL)**	2–6 years (range 3.6–5)	7 (range 3–11.9)	8.6 (range 4.8–22.1)	0.6334
**RBCs (10**^**6**^**/uL)**	7–12 years (3.8–4.8)	5.2 (range 3.1–8.1)	6 (range 3.5–10)	0.3136
**RBCs (10**^**6**^**/uL)**	13–18 years (0.4–5.2)	5.1 (range 4.6–5.5)	3.8 (range 1.2–6.1)	0.3524

*significant *p* < / = 0.05

### Patient characteristics during hospitalization

During hospitalization 5 males developed mild thrombocytosis (median number of PLTs: 607 × 10^3^/uL; range of values: 496–663 × 10^3^/uL) and an increase in inflammation measured in terms of high CRP levels (median of values: 2.55 mg/dL; range of values: 1.07–4.61 mg/dL). Three of these patients were less than 1 year; 1 patient was 2 years old and 1 patient was 12 years old. During hospitalization, patients less than 1-year-old manifested gastrointestinal symptoms and diarrhea; the 2-year-old patient had inflammation of the airways and pharyngitis and previously hospitalized for bronchiolitis; the 12-year-old patient developed salmonella gastroenteritis. On the basis of these data we can speculate that reactive thrombocytosis occurring in these patients may be due to an inflammatory process related to gastrointestinal disorders or inflammation of the airways.

### Patient treatments

There are no specific protocols to guide treatment of children with COVID-19. Analyzing the medical records we found that 20 patients (11 males and 9 female) did not receive any therapy (they were asymptomatic and apyretic); 18 patients (13 males and 5 females) were treated only with paracetamol as needed; 11 patients were treated with paracetamol and antibiotics (4 males and 7 females); 4 males and 2 females were treated only with antibiotics e 3 males only needed oxygen. Moreover, in addition to paracetamol and antibiotics, 2 females were treated with corticosteroid (they manifested bronchiolitis or cough); 1 female with anti-rheumatic drugs; 3 females with anti-inflammatory drugs and 2 females with heparin (they showed high values of inflammatory parameters and one of them was suffering from rheumatoid arthritis).

## Discussion

This study describes the characteristics of a sample of children admitted with COVID-19 to Bambino Gesù Children’s Hospital of Rome (Italy) in the period from the end of February to July 2020. All data were analyzed taking into account gender. In this retrospective study based on medical records data, we found that, compared to adults, the pediatric population gets less COVID-19 and had less severe clinical manifestations. Fever and cough were the dominant symptoms, while gastrointestinal symptoms were rare. Moreover, we found that all ages of childhood were susceptible to COVID-19: from a few days of life to 18 years. In particular, we found that females with COVID-19 were (although not significantly) older than males and required more days of hospitalization. Interestingly, from medical records we found lymphopenia in both sexes and high LDH values in females from 1 to 10 years old. LDH is a cytoplasmic enzyme present in all major organ systems and is released into the peripheral blood after cell death. Increased serum LDH levels are associated with pulmonary disease such as obstructive diseases, microbial pulmonary diseases and interstitial lung diseases such as acute respiratory distress syndrome [11].

During hospitalization some male patients developed mild thrombocytosis and exhibited increased inflammation evaluated in term of high CRP values. CRP is an inflammatory marker that plays an important role in host defense against invading pathogens [12]. Sun et al., shown that CRP was elevated in severe and critically adult patients with COVID-19 [13].

It has been found that in virus mRNA positive patients a decline of LDH in the serum correlated with viral mRNA elimination, suggesting that constitutive decrease of LDH levels probably predicts a favourable response. LDH can thus be used as indicators of disease progression [2].

Platelets have been increasingly recognized as an important component of the immune response to infections, an increase in their number above the normal range (thrombocytosis) has often been considered a sign of normal inflammatory reaction. Compared to primary thrombocytosis, the reactive thrombocytosis is not associated with higher risk of cardiovascular or thrombotic events [14].

## Conclusions

Compared to other pediatric studies on COVID-19, this retrospective study, in addition to evaluate clinical characteristics of children infected with SARS-CoV-2 in Italy, takes into account the sex of patients. Gender differences were highlighted only for some parameters. However, in order to have significant differences for the others parameters, the number of patients must be increased and further prospective studies should be conducted.

## Data Availability

The datasets used and/or analyzed during the current study are available from the corresponding author on reasonable request.

## Abbreviations

ACE2: Angiotensin-converting enzyme 2
CRP: C-reactive protein
EBV: Epstein-Barr virus
ESR: Erythrocyte sedimentation rate
HHV6: Herpes virus
LDH: Lactate dehydrogenase
PLTs: Platelets
RBCs: Red blood cells
RT-PCR: Real-time reverse transcription
SIP: Italian Society of Paediatrics
SITIP: Italian Society of paediatric Infectious Diseases
WBCs: White blood cells
